# Health System’s Responsiveness of Inpatients: Hospitals of Iran

**DOI:** 10.5539/gjhs.v7n7p106

**Published:** 2015-03-26

**Authors:** Mojtaba Mousavi Bazzaz, Majid Reza Erfanian Taghvaee, Maryam Salehi, Matin Bakhtiari, Zahra Abbasi Shaye

**Affiliations:** 1 School of Medicine, Mashhad University of Medical Sciences, Department of Community Medicine, Mashhad, Iran; 2 School of Medicine, Mashhad University of Medical Sciences, Department of Community Medicine and Research Center for Patient Safety, Mashhad, Iran; 3 Omolbanin Hospital, Mashhad University of Medical Sciences, Mashhad, Iran

**Keywords:** responsiveness, health system, hospital, inpatients

## Abstract

**Introduction:**

Additional to improving health and ensuring equitable financing that are two predominant goals of health system, another important goal of health systems is responsiveness to people’s non-medical expectations. In this study we try to assess the health system’s responsiveness in academic and non-academic hospitals.

**Methods:**

This is a cross sectional study done in summer 2014 in Mashhad-Iran, we surveyed a total number of 403 inpatients by multi-stage sampling. A questionnaire of responsiveness and a check list included demographic variables and characteristics of hospitalization were completed by trained interviewers. Scales from 0 to 10 was applied for each questionnaire at the end of assessment of questions.

**Result:**

403 participants Took part in this survey from 10 hospitals (6 academic and 4 non-academic hospitals). 124(30.8%) were from non-academic and 279(69.2%) from academic hospitals 140(34.7%) of patients were male and 263(65.3%) were female. mean age of participants was 36.77±1.52 years. The mean total score of responsiveness was 7.12±1.31 in academic hospitals and 6.99±1.38 in non-academic hospitals, considered as good performance. There was no significant difference between total scores of these two groups (p=0.38). Health care responsiveness score was higher in private (8.35±0.95) than other kinds of hospitals and charity hospitals had the lowest score (5.98±0.51).

**Conclusion:**

Responsiveness of health care system at hospitals is an important parameter for measuring patients’ perception of quality of health care. Although responsiveness rate of our hospitals are good but some components such as: choice health care providers, respect to autonomy of individuals, clear communication and confidentiality received lower responsiveness scores, therefore they require more attention and these domains can be the more significant choices that should be considered while designing improvement programs.

## 1. Introduction

The way that health systems interact with customers can affect their well-being and this interaction is considered as another goal; besides the improving health and ensuring equitable financing that are two goals of health system. Although some researchers have named this goal “patient experience”; World Health Organization (WHO) has termed this issue, health system responsiveness and has suggested evaluating the health system’s responsiveness parallel to the measurement of health system performance with more traditional indicators like mortality, morbidity (Gostin, Hodge, Valentine, & Nygren-Krug, 2003). Responsiveness reflects how the health system responds to the population’s legitimate non-medical expectations of health care (Bramesfeld, Klippel, Seidel, Schwartz, & Dierks, 2007). The concept of responsiveness has been consisted of eight domains according to Who framework: (1) respect for the dignity of persons; (2) autonomy to participate in health related decisions; (3) confidentiality and trust; (4) prompt attention; (5) adequate quality of basic amenities; (6) Clear communication; (7) access to social support networks (only for inpatients); and (8) choice of health care providers (Gostin et al., 2003).

The intrinsic goal of responsiveness is important because it deals with basic Human rights of individuals, reflects a positive orientation to those the system is designed to serve and holds promise to make health improvement among population. Patient satisfaction with non-medical aspects of care is often related to better compliance with treatment orders, prompt achievement of care and a better understanding and maintenance of medical information (Murphy, 1984; De silva, 2000). Achievements in the responsiveness domain lead to patients’ comfort. The greater the responsiveness of the health system, the higher will be the level of people’s comfort, irrespective of its impact on health (Gostin et al., 2003; De silva, 2000).

Improvements in responsiveness may come before changes in performance on either of the other two intrinsic goals. As it does not require a major investment, responsiveness can be improved much faster than health. Furthermore an important improvement in responsiveness does not necessarily entail a great investment in technology or staff as required for health promotion. Measuring responsiveness indicates which domain is more important from the individuals’ viewpoint and needs more solemn attention, in addition, information received from inequality distribution of responsiveness level can be used for guiding health systems’ resources to areas with inadequate services (Valentine, de Silva, Kawabata, Darby, Murray, & Evans, 2003).

In a survey performed in Southern Thailand to assess the perception of women who gave birth in a hospital about health system responsiveness, the majority of women (>80%) gave high ratings for dignity, clear communication, prompt attention and autonomy (Liabsuetrakul, Petmanee, Sanguanchua, & Oumudee, 2012). Hsu’s study assessed whether dimensions offered by WHO to measure responsiveness are applicable in evaluating the health system of Taiwan. Component analysis produced five factors (respect, access, confidentiality, basic amenities, and social support) that explained 63.5% of the total variances (Hsu, Chen, Hu, Yip, & Shu, 2006).

In world health report 2000, health system’s responsiveness of 191 members of WHO has been estimated and compared, using the questionnaires developed by WHO. In this report, health system’s responsiveness of Iran was in 100th place among 191 countries (World Health Report, 2000). So far only few studies were performed on responsiveness of health system in Iran and all of these studies reported moderate responsiveness scores in hospitals (Javadi, Karimi, Raiesi, Yaghoubi, & Kaveh, 2011; Ebrahimpour et al., 2013). An essential necessity of the assessment of health system’s responsiveness throughout the territory of Iran is perceived by health care professionals to promote the health of people. Healthy person is the fundamental of development and efforts to maintain and promote health, always considered as national priorities. The aim of this study was evaluation of health system proceedings by determining responsiveness level of non-medical expectations of inpatients in academic and non-academic hospitals.

## 2. Method

In this cross-sectional study that was done in summer 2014, we surveyed a total number of 403 patients that hospitalized in Mashhad, Iran. Mashhad is the second most populous city in Iran and is the capital of Razavi Khorasan Province. It is located in the North East of the country close to the borders of Afghanistan and Turkmenistan. Its population was 2,749,374 at the 2011 population census (Wikipedia).

Inclusion criteria was restricted to the patients with more 16 years of age or above and hospitalized in a ward at least 24 hours, exclusion criteria was hospitalization at emergency ward and ICU, unconsciousness and psychological disorders. Our sample size calculated according to estimation of a mean formula, we considered Ơ=0.58, z=1.96 and d=0.1 Ơ (Javadi et al., 2011). Population study included all inpatients in all academic and non-academic hospitals in Mashhad. Our sample study selected by multiple-stage sampling, first we divided all hospitals in two group of academic (Azad and Public) and non-academic (Private, charity). We selected 61% of samples from academic and 39% from non-academic hospitals proportional to total number of beds in each group. Each group also categorized according to total number of beds (beds>500, 500>= beds>150, 150>=beds), then hospitals in each category and required samples selected by random sampling. It is necessary to mention that because of no cooperation of some of non-academic hospitals to collect samples; finally we had 69.2% of samples from academic and 30.8% from academic hospitals.

Ethics Committee of Mashhad Medical Sciences University approved the study. Patients were given the necessary information about the research and were assured about the privacy of their personal data.

Azad hospitals referred to those under provision of non-public universities.

Data were collected at the bedside by trained interviewer using a checklist and 36 item questionnaire of responsiveness. We modified the Persian version of WHO questionnaire used in Javadi’s study (Javadi et al., 2011). To determine content validity, the questionnaire was given to 5 specialists of community medicine and medical health care, then it was examined according to criteria of relevancy; clarity, vague and simplicity of expression and desired reform was conducted. Cronbach’s alpha of the questionnaire was 0.89 for reliability. The checklist included basic variables (age, sex, educational level, duration and number of hospitalization, type of insurance and name of hospital ward).

The questionnaire consisted of 8 items of responsiveness: dignity (7 questions), autonomy (6 questions), confidentiality (2 questions), prompt attention (5 questions), quality of care (4 questions), communication (7 questions), access to social support networks (3 questions) and choice of health care providers (2 questions). In prompt attention domain we had questions about geographical access and urgency in addressing patients, in communication domain we considered questions about human and professional communication, and dignity domain consisted of questions about respect to dignity and unnecessary discrimination and inequality. We considered 0 to 10 score for each question and defined average 0-2.5 scores as very low responsiveness, 2.51-5 as low, 5.01-7.5 as good and 7.51-10 as very good responsiveness for each question. Finally the total mean score of each questionnaire was applied to scale of 0-10.

SPSS 11.5 software (SPSS Inc., Chicago, Illinois, USA) was used for all statistical analyses. Standard descriptive statistics were applied to describe the pattern of the data. Normality of the data was checked with 11032222E%%024965Kolmogorov–Smirnov test. Independent t and one-way ANOVA tests were applied to compare numerical data with normal distributions in two groups or more, respectively. Multivariate Linear regressions were used to predict factors influence on responsiveness scores. All tests were 2-tailed, and probability values 0.05 were considered significant.

## 3. Results

We had 403 participants in our research from 10 hospitals throughout Mashhad (6 academic and 4 non-academic hospitals). 124(30.8%) of patients were from non-academic and 279(69.2%) were from academic hospitals. The data showed that 140(34.7%) of participants were male and 263(65.3%) were female. mean age of participants was 36.77 years and 1.52 standard deviation with maximum of 85 and minimum of 16 years. Frequency distribution of patients’ characteristics separated by type of hospital is fully indicated in Table 1.

**Table 1:** Basic characteristics of study’s participants

Variables		Academic		Non-academic		Total
		Public	Azad	charity	private
Age (years)/mean±SD*		36.78±1.47	37.76±1.45	36.66±1.56	35.85±1.75	36.77±1.52
Sex/(N (%))	male	96(43.6)	8(13.6)	20(28.2)	16(30.2)	140(34.7)
	female	124(56.4)	51(86.4)	51(71.8)	37(69.8)	263(65.3)
Education(years)/(N (%))	illiterate	25(11.4)	5(8.6)	3(4.2)	1(1.9)	34(8.5)
	Non-academic	166(75.5)	46(79.3)	57(80.3)	34(64.2)	303(75.4)
	Academic	29(13.2)	7(12.1)	11(15.5)	18(34)	65(16.2)
DOH** (day)/mean±SD		6.38±4.07	3.02±2.84	3.05±1.06	2.24±1.59	4.80±2.74
NOH*** (day)/mean±SD		2.06±1.46	2.78±2.12	2.35±1.76	1.57±1.08	2.15±1.62
Insurance (N (%))	non	20(9.3)	2(3.4)	1(1.4)	4(7.7)	27(6.8)
	Social security	64(29.6)	46(78)	28(40)	19(36.5)	157(39.5)
	Health services	12(5.6)	4(6.8)	11(15.7)	22(42.3)	49(12.3)
	Armed forces	5(2.3)	0(0)	3(4.3)	2(3.8)	10(2.5)
	Iranian	68(31.5)	2(3.4)	0(0)	2(3.8)	72(18.1)
	rural	38(17.6)	0(0)	27(38.6)	1(1.9)	66(16.6)
	other	9(4.2)	5(8.5)	0(0)	2(3.8)	16(4)

* = standard deviation;

** = duration of hospitalization;

*** = number of hospitalization.

The mean overall score of responsiveness in all types of hospitals took part in this survey was 7.08±1.34; the mean total score, which was 7.12±1.31 in academic hospitals and 6.99±1.38 in non-academic hospitals. If we categorized hospitals in two groups by academic and non-academic, we didn’t have any significant difference between total scores of these two groups (p=0.38) but One-way ANOVA analysis showed that we had statistically significant difference among all of hospitals (Public, Azad, Charity and Private) in total score of responsiveness (p<0.001).

**Figure 1:**
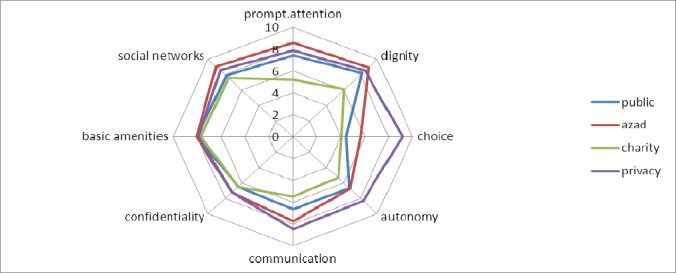
Comparing responsiveness domains of different kinds of hospitals

The lowest score was detected in choice of health care providers’ component (5.19±2.97) and the highest score related to access to social support networks’ component (8.08±1.81). Tables 2 and 3 depict the mean scores of different parts of health responsiveness separated by type of hospitals. The results showed that there were statistically significant differences between academic and non-academic hospitals in prompt attention, dignity and choice of provider components of health responsiveness (p<0.001), and academic hospitals had better performance in prompt attention and dignity items, while non-academic hospitals had better in choice of provider. Figure 1 schematically shows the scores in different areas of responsiveness in all type of hospitals. Overall, in prompt attention domain, geographically access questions received lower scores (7.04±2.88) than urgency addressing questions (7.38±2.41), in domain of communication; the score of professional relationship (6.54±2.25) was higher than human ones (7.59±2.52) and in component of dignity we found the lower score in questions related to Discriminatory and inequality behavior than respect to dignity (7.03±3.27 vs. 8.34±1.79).

**Table 2:** responsiveness domain scores in academic and non-academic hospitals

Responsiveness dimension	Academic	Non-academic	p-value (CI_95%_)
dignity	8.34±1.81	7.12±1.50	<0.001(-1.58,-0.85)
autonomy	6.68±1.62	6.59±1.73	0.59(-0.45,25)
confidentiality	6.63±2.96	6.79±2.15	0.58(-0.42,0.74)
prompt attention	7.65±2.31	6.32±1.88	<0.001(-1.79,-0.86)
Basic amenities	7.98±2.08	8.14±1.16	0.44(-0.24,0.54)
communication	6.88±2.18	6.75±1.98	0.56(-0.56,0.31)
social support	8.11±2.03	8.02±1.17	0.65(-0.47,0.29)
Choice of provider	4.72±2.91	6.23±2.84	<0.001(0.90,2.11)
Total score	7.12±1.31	6.99±1.38	0.38(-0.41,0.16)

**Table 3:** Responsiveness domain scores in different kinds of hospitals

Responsiveness component	Academic Public Azad		p-value (CI_95%_)	Non-academic Charity private		p-value (CI_95%_)
dignity	8.18±1.86	8.92±1.48	0.005(-1.26,-0.22)	6.07±0.64	8.54±1.09	<0.001(-2.78,-2.16)
autonomy	6.67±1.66	6.72±1.49	0.85(-0.49,0.40)	5.32±0.50	8.29±1.26	<0.001(-3.29,-2.64)
confidentiality	6.47±3.01	7.21±2.70	0.07(-1.55,0.07)	6.49±0.91	7.19±3.08	0.007(-1.46,0.07)
prompt attention	7.40±2.42	8.57±1.52	<0.001(-1.82,-.52)	5.19±0.79	7.84±1.84	<0.001(-3.13,-2.17)
Basic amenities	7.96±2.08	8.06±2.08	0.75(-0.70,0.51)	7.65±0.78	7.89±1.27	<0.001(-1.50,-0.74)
communication	6.65±2.23	7.71±1.78	0.001(-1.68,-0.44)	5.46±1.30	8.47±1.29	<0.001(-3.48,-2.54)
social support	7.86±2.14	9.04±1.14	<0.001(-1.76,-.62)	7.59±0.96	8.59±1.18	<0.001(-1.39,-0.60)
Choice of provider	4.46±2.85	5.69±2.94	0.005(-2.08,-0.38)	4.08±1.27	9.12±1.45	<0.001(-5.54,-4.55)
Total score	6.96±1.33	7.74±1.03	<0.001(-1.15,-0.41)	5.98±0.51	8.35±0.95	<0.001(-2.63,-2.11)

Our analysis indicated that women reported higher responsiveness rate rather than men (p<0.001, CI_95%_=-0.76,-0.21) and there was significant difference between hospital wards and score of responsiveness (p<0.001, CI_95%_=-1.09,-0.46) whereas the total score in surgery wards (7.25±1.29) were higher than internal wards (6.47±1.30). There was no significant difference among educational level, various kind of insurance coverage and total responsiveness score (p=0.17, p=0.89). We had poor reverse correlation between duration and number of hospitalization with total responsiveness score (p<0.001, r=0.30/p=0.01, r=0.12) but we didn’t find any correlation between age and responsiveness (p=0.14).

For detecting the predictors of the overall responsiveness score, a multiple linear regression model was done and demographic and hospitalization characteristics which had significant relation with the total responsiveness score entered to model. Sex, hospital wards and duration of hospitalization were significant predictors of responsiveness score (Table 4).

**Table 4:** Multiple linear regression of demographic and hospitalization characteristics on total responsiveness score

Predictor variables	β	p-value
Sex (male: reference)	0.40	0.01
Hospital ward (internal ward: reference)	0.44	0.02
DOH	-0.03	0.02
NOH	-0.06	0.17

## 4. Discussion

Health system responsiveness has been defined as one of the intrinsic goals of health care systems, with health promotion and equitable financing (Gostin et al., 2003; World Health Report, 2000). Responsiveness to patients’ non-medical expectations at present is defined as a key characteristic of effective health systems (Fazaeli, Ahmadi, Rashidian, & Sadoughi, 2014). In this survey, we aimed to investigate health system responsiveness from the perspectives of inpatients.

According to our results overall responsiveness of hospitals was 7.08±1.34, considered as good performance. This is similar to study Conducted in South Africa in which responsiveness score was 71 (range of 0-100), but higher than Turkish study with total score of 6.14 (range of 0-10) and Chinese study with score of 50-54 (range of 0-100) (Peltzer, 2012; Ugurluoglu & Celik, 2006; Kowal, Naidoo, Williams, & Chatterji, 2011).

There was not any significant difference between academic and non-academic hospitals and all hospitals were in good performance range. In category of academic hospitals, Azad hospitals have better performance scores compared to public hospitals and in non academic category, private hospital had better scores. Therefore; a reasonable explanation for similarity between academic and non academic hospital scores is that existence of two groups with different results in each category, modify the average score in the group. On the other hand it seems that establishment of Clinical Governance institution at most of hospitals, has a main role in promotion of public hospitals’ responsiveness (Clinical governance is a powerful, new and comprehensive mechanism for ensuring that high standards of clinical care are maintained and the quality of service is continuously improved) (Starey, 2001)

Policy makers and programmers should attend to patients’ experience especially from charity hospitals to improve health system responsiveness and it is necessary to establish the clinical governance institution in charity hospitals. according to our results, private hospitals with the total score of 8.35±0.95 had the best responsiveness among others; several surveys also indicated higher health care responsiveness in private than public (Peltzer, 2009; Mosallam, Aly, & Moharram, 2013; Rashidian, 2011; Malhotra & Do, 2013; Puentes-Rosas, Ruelas, Martínez-Monroy, & Garrido-Latorre, 2005; Adesanya et al., 2012). Given that patients must pay more charges at private hospitals, it is a reasonable expectation of people that private hospitals have greater responsiveness rate. Work forces, specialists and modern equipment and facilities of public hospitals are not lesser than private ones and these facilities provide potential capability for public hospitals to improve their responsiveness by proper utilization of them. Therefore; public hospitals can compete to private hospitals in terms of good service delivery.

As seen in this research, statistically difference between academic and nonacademic hospitals was found in three items: domains of prompt attention and dignity, which had higher scores in academic hospitals and domain of choice, with higher score in non-academics.

Totally, in this survey, the highest score was related to access to social support networks and quality of basic amenities which was consistent with other studies in Iran (Javadi et al., 2011; Ebrahimpour et al., 2013). Access to social support networks can be a key component for the improvement of adverse health characteristics among individuals (Gostin et al., 2003). In one study performed in Germany, however confidentiality is the best performing component in inpatient and outpatient care but access to social support also perform well, in addition, quality of health care in Peltzer’s study also had the highest perceived responsiveness score (Brumsfeld, 2007; Peltzer, 2012). In Egypt study, prompt attention considered as best responsiveness of health care (Mosallam et al., 2013).

The lowest score of responsiveness in our research was related to choice of provider component similar to Karami’s study (Karami-Tanha, Moradi-Lakeh, & Nojomi, 2014); authority of patients to choose the healthcare provider is considered to help improve patient access to care, as well as the quality of care (Gostin et al., 2003). Autonomy, confidentiality and communication also received low scores resembling two other studies that autonomy and communication had the lowest scores (Ebrahimpour et al., 2013; Mosallam et al., 2013). Autonomy is one of the elements of biomedical ethics, described as respecting viewpoints of patients and their optional choices.

This study showed statistically difference between responsiveness score given by men and women with higher scores among women, which is similar to some other surveys (Puentes-Rosas, 2005; Kowal, 2011; Peltzer, 2012); this difference it is probably due to the higher level of tolerance in women; however it is not consistent to some other studies (Ogurluoglu, 2006; Ebrahimpour et al., 2013).

Educational level, age, type of insurance coverage and number of hospitalization didn’t play a significant role in determining health care responsiveness; in German study there was no significant relationship between age and responsiveness but there was relationship between education and responsiveness; worse responsiveness was reported with less educated patients (Bramesfeld, Wedegärtner, Elgeti, & Bisson, 2007). Mosallam et al showed no differences between age and educational level with responsiveness score in their study (Mosallam et al., 2013). The total mean score of responsiveness of surgery wards were significantly higher than internal wards; it is probably due to overcrowding and lack of discipline at internal wards of Iran’s hospitals.

Providing a responsive system has the potential to lead to improved access and equity for all groups in the population, reduced delays in seeking health care and treatment, patient safety and quality assurance, patient satisfaction with health care, public image of health service, better use of resources, reduced failure to attend and readmission rates, communication and understanding of meanings between health consumers and providers resulting in Better compliance with recommended treatment, Clearer expectations, reduced medical errors and adverse events, Improved attendance at follow -up appointments, improved consumer satisfaction, reduced hospitalization rates (Stewar, 2006).

The main strength of our research is including major types of hospitals (public, azad, charity and private), not only public and private and also random sampling. However, there are some limitations in our study: first, the cross-sectional study design cannot establish the cause-effect relationship between health responsiveness and different variables. Second, we gathered self-reported subjective information rather than objective. Some of patients may have not declared their real idea about different components of responsiveness because they are afraid of negative effects on their received health care services. Third is a Hawthorne effect in which a study subject’s responses are changed as a result of the subject’s notice of being under observation; however we were tried to reduce this effect by emphasizing the independency of the interviewers. Because of low cooperation of male patients, most of our participants were female; it may be the reason of good responsiveness rate reported in our study, and it is the forth limitation. And finally, our results only limited to inpatients.

## 5. Conclusion

Responsiveness is an important parameter for measuring patients’ perception of quality of health care. We found that responsiveness rate of Mashhad-Iran hospitals are good but some items such as: giving patients opportunity to choose health care services (choice), respect to autonomy of individuals to participate fully in health-related decisions, providing health information to patients in language and format that furthers a patient’s understanding (communication) and considering privacy and confidentiality, received lower responsiveness scores, therefore they required more attention and these domains will be the priority area for improvement. Overall, health care responsiveness score was higher in private than other kinds of hospitals, and charity hospitals had the lowest score. Necessary efforts needed to promote responsiveness include, training and financial encouragement of staff, increasing knowledge and improving attitudes of patients and better allocation.
